# Evaluation of the construct validity of the Michigan Fatigability Index (MIFI) short forms: a cross-sectional survey study

**DOI:** 10.1186/s41687-026-01068-3

**Published:** 2026-04-16

**Authors:** Anna Kratz, Noelle Carlozzi, Susan Murphy, Tiffany Braley, Daniel Whibley, David Williams, Nora Fritz, Libak Abou, Michael Kallen

**Affiliations:** 1 Department of Physical Medicine and Rehabilitation, University of Michigan–Ann Arbor, Ann Arbor, USA; 2 Department of Neurology, University of Michigan–Ann Arbor, Ann Arbor, USA; 3 Department of Anesthesiology, University of Michigan–Ann Arbor, Ann Arbor, USA; 4 Wayne State University, Detroit, USA; 5 Northwestern University, Evanston, USA

**Keywords:** Multiple sclerosis, Fibromyalgia, Patient-reported outcome, Fatigability, Validity

## Abstract

**Background:**

The Michigan Fatigability Index (MIFI) is a patient reported outcome (PRO) measure that was developed in people with multiple sclerosis (MS) or fibromyalgia (FM). The MIFI consists of three items banks and corresponding 6-item short forms that assess physical fatigability, mental fatigability, or emotional fatigability. This report details examination of convergent and known-groups validity (i.e., construct validity) of the MIFI short forms for MS and FM.

**Methodology:**

A US web-based cross-sectional survey was distributed to *n* = 2650 participants (FM *n* = 1242; MS *n* = 1408) who completed the MIFI measure and measures of fatigue intensity, severity, impact, fatigability, physical and cognitive functioning, anxiety, and depressive symptoms to establish convergent validity. Tests of known groups validity were based on disease severity scores for MS and FM and PROMIS measures of global physical and mental health scores, which were dichotomized into upper and lower tertile health groups.

**Results:**

Convergent validity of the MIFI short forms was supported by moderate to high correlations (r = > 0.60) with measures of similar constructs. Each MIFI fatigability short form was able to distinguish between lower and upper tertiles on global physical and mental health with large effects (η²>0.3), and between lower and upper disease severity subgroups with small to large effects, supporting known groups validity.

**Conclusions:**

Results support the construct validity of the MIFI physical fatigability, mental fatigability, and emotional fatigability short forms for MS and FM. The MIFI measures, which are brief and developed specifically for use in clinical populations with high fatigue burden, should be considered for use in clinical and research applications.

## Introduction

Chronic conditions such as multiple sclerosis (MS; an autoimmune mediated neurological condition that affects 1 million people in the United States (US)) [1] and fibromyalgia (FM; a condition characterized by widespread body pain and physical, emotional, and cognitive symptoms affecting 6.4% of adults in the US) [2, 3] are characterized by fatigue, a subjective feeling of lack of energy, tiredness, and/or exhaustion [2, 4–6]. Due to its significant impact on individuals’ health and well-being, fatigue is often the focus of clinical research within these populations. However, efforts to mitigate the effects of fatigue across conditions are hindered in part by current assessment methods. For example, existing measures fail to consider the intensity of perceived fatigue in the context of a person’s activity and functioning, which is critical to accurately quantify the extent to which fatigue is problematic for a person.

To address this issue, one potential solution is to consider the construct of perceived fatigability, which reflects subjective fatigue intensity in relation to activity levels [7–9]. Fatigability has been defined as a distinct construct from fatigue [10, 11] and conceptualized as having both subjective (perceived fatigability) and objective components (performance fatigability) [12]. In contrast to performance fatigability, which requires performance-based tests to quantify, perceived fatigability can be measured through patient-reported outcome measures (PROs), making assessment more accessible across clinical and research settings. Despite the recent growing attention on fatigability research, mostly among older adults [13–15], very few studies have been conducted to enhance our understanding of this construct in chronic conditions where fatigue is common, such as MS or FM [16–18]. Robust measures of perceived fatigability are needed for use in conditions such as MS and FM because assessment of fatigue intensity alone provides a narrow and limited clinical picture. For example, functional and activity levels may be quite different between patients reporting the same fatigue intensity score [7]. In addition, failing to account for how activity impacts fatigue limits the ability to assess the effects of treatments, such as physical therapy guided exercise. These considerations are very important for neurological and pain conditions where functional outcomes of treatment are often considered alongside symptom severity outcomes [19].

To address this knowledge gap and build upon available measures of fatigability, our group has conducted a series of studies. In a previous qualitative study that included participants with MS or FM diagnosis or general population adults across the adult life span (aged 18 years and older without MS or FM) [20], we developed a person-centered conceptual model of perceived fatigability that highlighted the need for measures of three distinct fatigability subdomains: physical, mental, and emotional fatigability. The person-centered conceptual model [20]served as a theoretical foundation to develop a comprehensive PRO of fatigability called the Michigan Fatigability Index (MIFI) [21]. The MIFI, unlike previous self-reported measures of fatigability such as the Situational Fatigue Scale (SFS) [22] and the Pittsburgh Fatigability Scale (PFS) [15], includes a range of levels of intensity of physical, cognitive, and social/emotional activities, which allows for assessment of a broader range of fatigability severity. In a recent development paper [23], we presented a report of the process to develop and calibrate three item banks and associated short forms to assess physical, mental, and emotional fatigability of the MIFI. This paper also highlighted preliminary data indicating that the MIFI computer adaptive tests and short forms demonstrate excellent reliability [23].

Before recommending the use of the newly developed MIFI in clinical practice and research, it is imperative to confirm its validity, or the degree to which it measures the construct it purports to measure [24]. Therefore, the aim of the current report is to evaluate convergent and known-groups validity (i.e., construct validity [25, 26]) of the MIFI short forms in a sample of adults with either MS or FM.

## Methods

### Participants and study procedures

Survey data were collected from a total of 2650 participants with fibromyalgia (*n* = 1242) or multiple sclerosis (*n* = 1408). The Medical Institutional Review Board at the University of Michigan reviewed all study procedures and deemed this study met federal and institutional criteria for exempt human subjects research. As detailed in the measure development paper [23], surveys were programmed into Qualtrics, an online research tool that allows for direct data entry by study participants and provides real-time data export and automated accrual report features [27]. Qualtrics study links were disseminated through emails advertising the study; emails were sent by the National Multiple Sclerosis Society and the National Fibromyalgia Association to their respective listservs. Individuals who completed the survey had the option of entering a raffle to win a $100 gift card (20 winners within each diagnostic group). Inclusion criteria for the survey study were: 18 years of age or older, and self-reported diagnosis of FM and/or MS; survey questions were used to assess veracity of diagnosis report, such as reporting use of a disease modifying therapy (for MS) and scores on the American College of Rheumatology 2016 Fibromyalgia (FM) Survey Criteria [28–30] (for FM). For this study, we aimed to collect at least 1000 cases with self-reported FM and/or MS within the United States. Data were collected between May 12, 2021 and June 28, 2021 for the MS sample and between July 2, 2021 and July 14, 2021 for the FM sample.

### Measures

*Perceived Fatigability* was assessed with the measure under evaluation, the Michigan Fatigability Index (MIFI; see [23] for development details). The MIFI consists of three measures that assess fatigability by asking respondents to rate the fatigue intensity theywould expect to develop when engaging in various activities on a response scale of 1 (not at all) to 5 (very much). There were 42 items that assess physical fatigability, 28 items that assessed mental fatigability, and 23 items that assessed emotional fatigability. For each subdomain, items are summed and converted to a T score (Mean = 50, SD = 10). Perceived fatigability was also assessed with the Pittsburgh Fatigability Scale (PFS) [31] a 20-item survey to measure level of physical and mental fatigue (on a numerical rating scale from 0 “no fatigue” to 5 “extreme fatigue”) that the respondent feels or would expect to feel after doing a particular activity (e.g., brisk or fast walk for 1 h). Each item is answered in relation to physical fatigue and mental fatigue regardless of whether the participant has done the activity within the reporting timeframe of 1 month. Both physical and mental fatigability subdomains measured with the PFS range from 0 to 50, with higher scores indicating more fatigability. The PFS was selected to evaluate the convergent validity of the MIFI scales because it has been validated in clinical populations including MS [18, 32–34] and is a commonly used self-report measure of perceived fatigability, primarily in clinical research of older adults [35].

*Fatigue Intensity and Interference* were assessed with the PROMIS Fatigue_FM_ Profile short form [36], which consists of four 4-item subscales: fatigue experience (“intensity”) and fatigue interference across three subdomains – social impact, motivational impact, and cognitive impact of fatigue. Subscale total scores reflected sums of all item responses, which are then converted to a general population reference T score (Mean = 50, SD = 10).

*Fatigue Severity* was assessed with the Fatigue Severity Scale (FSS) [37], a 9 item self-report measure of fatigue severity; most items focus on physical fatigue or the impact of fatigue on physical function. Summed FSS scores range from 9 to 63; higher scores indicate more fatigue with scores of 36 or more indicating clinically important fatigue.

*Perceived Cognitive Dysfunction* was assessed with the 20-item Perceived Deficits Questionnaire (PDQ) [38], which asks respondents to rate how often they experienced different types of perceived cognitive dysfunction in the past 4 weeks, on a scale from 0 (never) to 4 (almost always). The PDQ assesses difficulties in four cognitive domains: attention, retrospective memory, prospective memory, and planning and organization (i.e., executive function). The total score for the PDQ is the sum of the responses on the 20 items and higher scores indicate greater perceived cognitive dysfunction.

*Physical Functioning* was assessed with the PROMIS Physical Function Short Form 8b [39–41], which assess ability to engage in eight activities, rated on two scales reflecting level of difficulty (5 “without any difficulty” to 1 “unable to do”) or limits in function (5 “not at all” to 1 “cannot do”). Total sum scores are converted to T Score (Mean = 50, SD = 10) with higher scores indicating higher physical function.

*Anxiety* was assessed with the PROMIS Anxiety Short Form 8a [42] that assesses frequency of eight anxiety symptoms in the past seven days on a scale from 1 (never) to 5 (always). Summed scores are converted to a T Score (Mean = 50, SD = 10) where higher scores indicate greater anxiety symptoms severity.

*Depressive Symptom Severity* was assessed with the PROMIS Depression Short Form 8a that assesses frequency of eight depressive symptoms in the past seven days on a scale from 1 (never) to 5 (always). Summed scores are converted to a T Score (Mean = 50, SD = 10) where higher scores indicate greater depressive symptoms severity.

*Global Physical Health and Global Mental Health* were assessed with the PROMIS Global Health measure [43], which includes 10 items that measure health across the physical, mental, and social domains. Summed scores are converted to a T Score scale (Mean = 50, SD = 10) where higher scores indicate greater health for either the physical or mental global domain.

*MS Disease Severity/Disability* was assessed with the Patient Determined Disability Steps (PDDS) [44], a validated [45] single-item scale that requires respondents to rate their level of MS disability on a scale from 0 (normal) to 8 (bedridden).

*FM Disease Severity* was assessed with the American College of Rheumatology (ACR) fibromyalgia survey criteria [30, 46] which assesses the number of painful body regions using the Michigan Body Map (0–19) and includes questions about related symptoms such as problems with thinking, fatigue, and sleep (0–12). A resulting continuous total score (possible range 0–31) is used to quantify “fibromyalgianess” or the severity of FM.

#### Data analysis

Construct validity of the MIFI subscales was assessed by examining convergent validity through Pearson correlations between the MIFI and relevant measures and through conducting known groups analyses.

##### Convergent validity

Selection of variables to explore convergent validity for the three MIFI short forms was driven by a number of assumptions. First, that the MIFI subdomain scores and short forms are distinct but share some conceptual overlap with one another and with other measures of fatigability (e.g., the PFS). The MIFI physical fatigability short form was expected to have strong correlations with physical fatigue and functioning variables (PROMIS fatigue experience, FSS, and PROMIS physical function). The MIFI mental fatigability short form was expected to have strong correlations with mental and motivational variables (PROMIS fatigue cognitive impact, PROMIS fatigue motivational impact, and the PDQ). The MIFI emotional fatigability short form was expected to have strong correlations with motivational and emotional variables (PROMIS measures of fatigue motivational impact, depression, and anxiety). Our a priori criteria for convergent validity was that correlations between the MIFI short forms and similar constructs should be high (i.e., ≥ 0.60) [47].

##### Known-group validity

We also evaluated the ability of short forms to discriminate between subgroups of participants expected to differ on fatigability assessment. We conducted known groups analyses on the combined sample as well as within each subsample. For each of the MIFI subdomains, we compared mean fatigability short form score differences across tertile groups for PROMIS Global Physical Health and PROMIS Global Mental Health [43]. We hypothesized that those individuals with better health status (for both physical and mental health) would have less fatigability for each of the MIFI subdomains than those individuals reporting worse health status.

For the FM subsample known groups analyses, we compared mean fatigability short form score differences across median split (low versus high) on the ACR FM survey criteria, which provides a continuous index of severity of FM, called “fibromyalgianess”. We hypothesized that those low in fibromyalgianess would have low levels of fatigability across all MIFI domains compared to those with high fibromyalgianess.

For the MS subsample known groups analyses, we compared mean fatigability short form score differences across disability severity groups of low disability (PDDS scores 0–1) versus moderate to high disability (PDDS scores 2–8). We hypothesized that those low in MS disability would have lower levels of fatigability across all MIFI domains compared to those with high disability.

For all known groups analyses, we used one-way analysis of variance (ANOVA) and examined η² as our group difference effect size measure and employed the following interpretation guidelines: “small” effect is *η²*=0.01–0.05; “medium” effect is *η²*=0.06–0.14; and “large” effect is *η²*=>0.14 [48].

## Results

In total, 2650 participants (fibromyalgia *n* = 1242; multiple sclerosis *n* = 1408) were included in the validation study, with a mean age of 54.5 years (age range = 20–88 years), 89.5% female, 90% White, and 92.9% with more than a high school education. See Table 1 for additional details regarding sample demographic and clinical characteristics.

**Table 1:** Sample descriptive statistics on demographic, clinical and key study variables

	Total Sample (*N* = 2650)	Fibromyalgia (*n* = 1242)	Multiple Sclerosis (*n* = 1408)
Age, Mean ± SD (years)	54.49 ± 13.11	60.18 ± 11.17	49.14 ± 12.55
Sex (%)			
Male	10.5	3.9	16.7
Female	89.5	96.1	83.3
Ethnicity (%)			
Hispanic	5.6	4.9	6.3
Non-Hispanic	89.4	88.7	90.2
Unknown/Not Reported	5.0	6.4	3.5
Race (%)			
White	90.0	92.6	87.5
Black	4.8	2.4	7.1
Asian	0.8	0.9	0.7
Native American	0.4	0.5	0.4
Bi-/Multi-racial	2.6	2.9	2.3
Missing/Unknown	1.4	0.8	2.1
Education Level			
High school or less	7.1	8.8	5.6
Some College	27.3	31.9	22.8
College Degree	35.6	34.9	36.2
Advanced Degree	30.0	24.5	35.4
MS Subtype			
Relapsing Remitting	NA	NA	77.6
Progressive Subtype	NA	NA	17.4
Unknown/Missing	NA	NA	5.0
	Mean ± SD (years)	Mean ± SD (years)	Mean ± SD (years)
Michigan Fatigability Index			
Physical fatigability SF T-Score	50 ± 10	53.76 ± 8.27	46.69 ± 10.00
Mental fatigability SF T-Score	50 ± 10	53.02 ± 8.82	47.30 ± 9.89
Emotional fatigability SF T-Score	50 ± 10	53.01 ± 8.83	47.30 ± 9.86
Pittsburgh Fatigability Scale			
Physical Scale	29.10 ± 10.85	33.27 ± 8.84	25.33 ± 11.13
Mental Scale	21.60 ± 10.86	25.85 ± 12.31	17.76 ± 12.70
PROMIS Fatigue Profile			
Experience T-Score	62.07 ± 9.36	65.61 ± 7.94	58.86 ± 9.40
Social Impact T-Score	61.83 ± 9.58	65.66 ± 7.94	58.39 ± 9.50
Motivational Impact T-Score	62.45 ± 9.35	65.83 ± 8.19	59.39 ± 9.29
Cognitive Impact T-Score	63.31 ± 9.45	66.38 ± 8.04	60.55 ± 9.77
Fatigue Severity Scale	49.03 ± 13.51	54.19 ± 10.23	44.34 ± 14.39
Perceived Deficits Questionnaire	35.14 ± 16.39	38.87 ± 14.61	31.73 ± 17.17
PROMIS Physical Function T-Score	38.77 ± 7.98	35.53 ± 5.27	41.73 ± 8.85
PROMIS Anxiety T-Score	55.98 ± 10.05	58.55 ± 9.06	53.63 ± 10.34
PROMIS Depression T-Score	55.72 ± 10.16	58.91 ± 9.12	52.80 ± 10.19
PROMIS Pain Intensity T-Score	59.92 ± 9.88	65.21 ± 6.48	55.05 ± 9.98

Note. SD = standard deviation; MIFI = Michigan Fatigability Index; SF = short form; PROMIS = Patient Reported Outcomes Measurement Information System

Correlations between the three fatigability short form scores and other select measures provide considerable evidence for construct validity for each of the short forms (Table 2). For the combined sample and both subsamples, correlations *r* ≥ 0.60 were observed between all MIFI short forms and measures of similar constructs, except for the MIFI emotional fatigability short form. The MIFI emotional fatigability short form demonstrated correlations above the ≥ 0.60 threshold for all similar constructs except for with the PFS physical and mental fatigability subscales (range *r* = 0.54- *r* = 0.58).

**Table 2:** Pearson correlation coefficients examining convergent validity of the MIFI physical fatigability, mental fatigability, and emotional fatigability 6-item short forms for the combined sample and FM and MS subsamples (all *p* < 0.001)

	*Physical Fatigability*				
	PFS-Physical Fatigability	PFS-Mental Fatigability	*PROMIS Fatigue experience*	*FSS*	*PROMIS Physical Function*
Combined Sample	0.85	0.71	0.62	0.70	-0.84
FM Subsample	0.78	0.66	0.61	0.64	-0.79
MS Subsample	0.73	0.69	0.60	0.70	-0.84
	*Mental Fatigability*				
	PFS-Physical Fatigability	PFS- Mental Fatigability	PROMIS Fatigue cognitive impact	PROMIS Fatigue motivational impact	PDQ Perceived Cognitive dysfunction
Combined Sample	0.64	0.66	0.60	0.78	0.76
FM Subsample	0.60	0.66	0.73	0.57	0.70
MS Subsample	0.60	0.65	0.78	0.62	0.77
	*Emotional Fatigability*				
	PFS-Physical Fatigability	PFS- Mental Fatigability	PROMIS Fatigue motivational impact	PROMIS Anxiety	PROMIS Depression
Combined Sample	0.56	0.58	0.69	0.68	0.62
FM Subsample	0.54	0.57	0.63	0.64	0.65
MS Subsample	0.57	0.58	0.64	0.68	0.62

Note. MIFI = Michigan Fatigability Index; PFS = Pittsburgh Fatigability Scale; PROMIS = Patient Reported Outcomes Measurement Information System; FSS = Fatigue Severity Scale; PDQ = Perceived Deficits Questionnaire; FM = fibromyalgia; MS = multiple sclerosis

Scores from our three new fatigability short forms discriminated between known participant subgroups in expected ways for PROMIS Global: Mental Health, PROMIS Global: Physical Health (Table 3), ACR FM Survey criteria (Table 4; FM severity), and PDDS scores (Table 5; MS severity). For each targeted grouping variable, the “worse” category/those reflecting worse health status, had significantly worse mean scores for each of the three fatigability short form scores. Effect sizes for the known groups analyses of the MIFI short forms were large for the physical fatigability short form, were medium to large for the mental fatigability short form and were small to large for the emotional fatigability short form.

**Table 3:** Known groups analyses conducted in the combined sample comparing MIFI Fatigability T-Scores between top and bottom tertiles on the PROMIS Global Physical Health (top of table) and PROMIS Global Mental Health (bottom of table)

MIFI Measure	PROMIS Global Physical Health	*N*	Mean	SD	*p* value	η²
Physical Fatigability T-Score	Lower tertile	798	58.6	6.7	*< 0.001*	0.627 ***
	Upper tertile	751	40.8	7.0		
Mental Fatigability T-Score	Lower tertile	822	56.9	8.2	*< 0.001*	0.396 ***
	Upper tertile	773	43.5	8.3		
Emotional Fatigability T-Score	Lower tertile	821	55.	8.9	*< 0.001*	0.304 ***
	Upper tertile	774	44.2	8.7		
**MIFI Measure**	**PROMIS Global** ** Mental Health**	**N**	**Mean**	**SD**	***p*** **value**	**η²**
Physical Fatigability T-Score	Lower tertile	782	57.6	7.4	*< 0.001*	0.471 ***
	Upper tertile	755	42.6	8.6		
Mental Fatigability T-Score	Lower tertile	813	56.6	8.4	*< 0.001*	0.406 ***
	Upper tertile	775	42.8	8.3		
Emotional Fatigability T-Score	Lower tertile	813	55.7	8.9	*< 0.001*	0.316 ***
	Upper tertile	775	43.6	8.8		

Note. * = small effect (≥ 0.01 to 0.05); ** = medium effect (≥ 0.06 to 0.13); *** = large effect (≥ 0.14); MIFI = Michigan Fatigability Index; PROMIS = Patient Reported Outcomes Measurement Information System

**Table 4:** Known groups analyses in the FM subsample comparing MIFI Fatigability T-Scores between low (mild FM severity) and high (moderate/severe FM severity) scores on the ACR fibromyalgia survey criteria based on median split

MIFI Measure	ACR FM Survey Score	*N*	Mean	SD	*p* value	η²
Physical Fatigability T-Score	Low	460	50.7	7.1	*< 0.001*	0.141 ***
	High	505	56.7	8.0		
Mental Fatigability T-Score	Low	475	49.6	8.1	*< 0.001*	0.119 **
	High	520	55.7	8.5		
Emotional Fatigability T-Score	Low	475	50.2	8.2	*< 0.001*	0.084 **
	High	520	55.3	8.5		

Note. * = small effect (≥ 0.01 to 0.05); ** = medium effect (≥ 0.06 to 0.13); *** = large effect (≥ 0.14); MIFI = Michigan Fatigability Index; ACR = American College of Rheumatology; FM = fibromyalgia

**Table 5:** Known groups analyses in the MS subsample comparing MIFI Fatigability T-Scores between those with no or mild disability (PDDS scores 0–1) or moderate to high disability (PDDS scores 2–8)

MIFI Measure	PDDS Score	*N*	Mean	SD	*p* value	η²
Physical Fatigability T-Score	0–1	481	40.0	7.6	*< 0.001*	0.368 ***
	2–8	601	52.0	7.9		
Mental Fatigability T-Score	0–1	488	44.5	9.0	*< 0.001*	0.070 **
	2–8	618	49.7	9.8		
Emotional Fatigability T-Score	0–1	488	45.0	9.3	*< 0.001*	0.044 *
	2–8	618	49.2	9.9		

Note. * = small effect (≥ 0.01 to 0.05); ** = medium effect (≥ 0.06 to 0.13); *** = large effect (≥ 0.14); MIFI = Michigan Fatigability Index; PDDS = Patient Determined Disease Steps; MS = multiple sclerosis

## Discussion

The aim of this paper is to present initial evidence of the construct validity of the newly developed MIFI short forms in a large sample of individuals with FM or MS. There was good evidence for the convergent validity of the MIFI measures. For the MIFI physical fatigability and mental fatigability short forms in particular, there were clear patterns of strong correlations (*r* > 0.60) with conceptually similar measures. The MIFI emotional fatigability short form also showed strong correlations with similar constructs but fell just short of meeting the a priori *r* ≥ 0.60 threshold criteria for convergent validity with the current legacy measure of physical and mental fatigability (range *r* = 0.54 – *r* = 0.58), though these slight deviations from the a priori threshold do not suggest the short form is invalid. Known groups analyses demonstrated that all three MIFI short forms distinguished between upper and lower tertiles of global physical and mental health with large effect sizes. And, within the diagnostic subgroups, the MIFI short forms distinguished between low and high disease severity. This evidence of good construct validity for the MIFI measures is added to findings of other positive psychometric properties presented in the companion paper [23]; these include very low ceiling and floor effects (all < 1%) and excellent measurement reliability (> 0.70 for most of the measurement range) and internal consistency reliability (all α > 0.90). Together, these companion papers are the start of what is intended to be a robust body of research exploring the utility of the MIFI PRO in fatigued clinical populations.

Existing measures of perceived fatigability, such as the Pittsburgh Fatigability Scale [15] and the Situational Fatigue Scale [22] predate the creation of the MIFI. The strong correlations observed between the MIFI Physical Fatigability short form and the Pittsburgh Fatigability Scale physical subscale may raise questions about potential redundancy between these scales. However, development of the MIFI was undertaken to address limitations of these existing scales, including focus on fatigue in the context of a narrow range of activity. In particular, the other measures feature a lack of assessment of fatigue in the context of mentally strenuous and low intensity physical activities, which are more likely to be seen in those with high disease burden and/or disability. The fact that these other measures seem to focus on characterizing fatigability on the low end of the severity spectrum raises the possibility that these existing measures will not be useful in clinical populations characterized by high levels of fatigability. Consistent with previous models and measures of fatigability and our stakeholder input, the MIFI assesses the experience of physical fatigability and mental (“cognitive”) fatigability. The MIFI includes items that assess fatigue in the context of a broad range of mentally-fatiguing and physically fatiguing activities and situations, with the intention that the measure can be useful across a wide range of fatigability severity. Another novel contribution of the MIFI is the inclusion of a measure of emotional fatigability, which was defined as developing a sense of being emotionally overwhelmed, empty, exhausted, defeated, “burned out”, or of “having nothing left to give” [20]. This novel construct and measure showed strong evidence of convergent validity with measures of motivational impact of fatigue, anxiety, and depression. Although correlations of the MIFI emotional fatigability short form with the PFS physical and mental fatigability scales fell just short of the prespecified criteria to support convergent validity, this is not entirely surprising given that the PFS does not have a subscale for emotional fatigability. Indeed, emotional fatigability is a distinct and underexplored facet of fatigability that lacks a “gold standard” legacy measure that would serve as a good comparator for evaluation of convergent validity. These distinctions suggest that while the PFS and MIFI are related, the MIFI provides a more comprehensive and multidimensional assessment of perceived fatigability.

The MIFI short forms may have meaningful applications in both clinical and research settings. Clinically, multidimensional fatigability profiling may assist healthcare providers in tailoring rehabilitation strategies, such as exercise-based interventions where understanding activity-specific fatigue responses is essential. In research contexts, the MIFI may serve as a sensitive PRO in clinical trials targeting fatigue and as a phenotyping tool to identify subgroups with distinct fatigability patterns.

### Study strengths and limitations

This study had several strengths, including a large sample collected from across the United States and comprehensive evaluation of multiple aspects of fatigue as well as other relevant health-related quality of life domains (e.g., physical functioning, mood). Limitations of the study include a study sample that has limited diversity in terms of race, education level, sex, and clinical diagnosis. Recruitment through advocacy organizations could have further limited generalizability of the sample to broader populations. Generalizability may also have been affected by the fact that clinical diagnosis was collected via self-report and was not verified via record confirmation. Future replication work in a medically verified sample is potentially warranted. Furthermore, the cross-sectional study design precluded evaluation of some important psychometric qualities, including test-retest reliability and responsiveness and sensitivity to change. Addition work in longitudinal samples is needed.

### Future directions

Though these data supporting the construct validity of the MIFI short forms are necessary to establish the clinical utility of these measures, further work is needed to establish other types of validity as well as other psychometric characteristics (test-retest reliability, responsiveness and sensitivity to change, etc.) of the MIFI. Work to establish the validity of these measures in other clinical populations is also needed.

Tertile- and median-splits on scales of mental and physical health, fibromyalgia severity, and MS-related disability were used to allow for examination of known-groups validity of the MIFI scales in terms of clinically meaningful groupings. Future studies may complement this approach with regression-based analyses using continuous scores on these measures of health and disease severity.

The observed correlational patterns, where correlations between theoretically aligned constructs were consistently stronger than those between conceptually distinct constructs, provides preliminary support for discriminant validity. However, future research employing multitrait–multimethod matrix (MTMM) [47] design or structural equation modeling are needed to strengthen evidence of discriminant validity.

Because the MIFI subscales and several comparator measures were derived from the PROMIS framework, some degree of conceptual and methodological overlap may have inflated the magnitude of correlations in convergent analyses. Although this does not undermine the validity findings, future investigations may benefit from incorporating non-PROMIS comparator measures or conducting multivariate analyses to examine construct independence and potential multicollinearity among related PROs.

## Conclusions

The MIFI provides a comprehensive assessment of perceived physical, mental (“cognitive”), and emotional fatigability. Finding support the construct validity of these measure in a combined sample of individuals with FM or MS. Because the validation sample included individuals with one of two common clinical conditions characterized by severe fatigue and fatigability, findings may be generalizable to other populations with high fatigue burden. The MIFI may be used in clinical settings to phenotype patients and plan and track treatment strategies or in research settings as a clinical trial outcome measure.

## Data Availability

The datasets used and/or analyzed during the current study are available from the corresponding author on reasonable request.
